# *Toxoplasma gondii* Investigation of Home-Reared Pigs through Real-Time PCR and Digital Droplet PCR: A Very Low Prevalence

**DOI:** 10.3390/pathogens12070882

**Published:** 2023-06-27

**Authors:** Claudio de Martinis, Alessia Pucciarelli, Maria Ottaiano, Roberta Pellicanò, Loredana Baldi, Vincenzo Veneziano, Giovanni Sgroi, Federica Boccia, Carmine Carbone, Lorena Cardillo, Giovanna Fusco

**Affiliations:** 1 Unit of Exotic and Vector-Borne Diseases, Department of Animal Health, Istituto Zooprofilattico Sperimentale del Mezzogiorno, 80055 Naples, Italy; claudio.demartinis@izsmportici.it (C.d.M.); alessia.pucciarelli@izsmportici.it (A.P.); 2 Regional Observatory of Epidemiology and Biostatistic, Istituto Zooprofilattico Sperimentale del Mezzogiorno, 80055 Naples, Italy; maria.ottaiano@izsmportici.it (M.O.); roberta.pellicano@izsmportici.it (R.P.); loredana.baldi@izsmportici.it (L.B.); 3 Dipartimento di Medicina Veterinaria e Produzioni Animali, 80137 Napoli, Italy; vinvene@unina.it; 4 Unit of Wildlife, Department of Animal Health, Istituto Zooprofilattico Sperimentale del Mezzogiorno, 80055 Naples, Italy; giovanni.sgroi@izsmportici.it; 5 Azienda Sanitaria Locale (ASL) Napoli 3 Sud, Unit of Animal Health, Department of Prevention, 80059 Naples, Italy; f.boccia@aslnapoli3sud.it (F.B.); c.carbone@aslnapoli3sud.it (C.C.); 6 Unit of Virology, Istituto Zooprofilattico Sperimentale del Mezzogiorno, 80055 Naples, Italy; giovanna.fusco@izsmportici.it

**Keywords:** *Toxoplasma gondii*, pig, molecular biology, biosecurity

## Abstract

*Toxoplasma gondii* is a widespread protozoon that can infect both animals and humans. The main route of human infection is the consumption of the raw or undercooked meat of several animal species, including pigs. Although *T. gondii* represents a public health concern, control during slaughter is not mandatory, leading to a lack of information on the impact on human contagion as well as poor data availability in domestic animals intended for human consumption. We studied the presence of *T. gondii* in home-reared pigs, an unconventional type of farming subjected to stringent breeding conditions dictated by Italian regulation. Thus, the diaphragms, livers and masseter muscles from 480 pigs in Napoli Province (Italy) were analyzed using real-time PCR and digital droplet PCR. The results showed four matrices that tested positive for *T. gondii* with very low protozoan loads (0.62%), belonging to three different animals. The low density of the animals (the maximum was four animals per farm) and the biosafety farming features decisively contributed to the bioexclusion of this pathogen. Comparing these results to intensive and extensive farm data, lower exposure to the parasite was revealed, suggesting that this farming method might mitigate the risk of human exposure through meat consumption.

## 1. Introduction

*Toxoplasma gondii* is an obligate intracellular protozoon with zoonotic potential, which is responsible for toxoplasmosis. While this parasite has a wide host range, the definitive host is represented by members of the *Felidae* family [1]. 

The *T. gondii* life cycle is characterized by asexual reproduction that occurs in intermediate hosts, such as mammals, including humans and birds, which can act as a reservoir, and sexual reproduction, which occurs in felids at the gut level. The definitive host can excrete large amounts of oocysts with feces and contaminate soil, water and feed, which can represent a source of infection for intermediate hosts [2].

In humans, the infection mainly occurs through the consumption of raw or undercooked meat, vegetables and water contaminated by oocysts [3,4]. 

Human toxoplasmosis, in most cases, runs asymptomatically or with flu-like symptoms, but it can be responsible for severe illness in young or immunocompromised individuals, who can experience toxoplasmatic encephalitis, myocarditis and pneumonia. In pregnant women, a vertical transmission can occur, which is responsible for abortion, stillbirth and fetal developmental disorders, thus representing a global threat [5].

Due to the lack of symptoms or the poor symptoms that the protozoon generally causes, the infection is often underdetected and undereported [6], unless the disease occurs during pregnancy. Therefore, the data mainly belong to women of childbearing age, where seroprevalence ranges from 6% to 80% [7]. A study conducted in 2000, involving several centers in Europe, including two Italian centers located in Naples and Milan, indicated that diet is the main risk factor in pregnant women, as the intake of undercooked meat is responsible for 30% to 63% of all cases [8]. In particular, pork seems to be one of the main cause of *T. gondii* infection, and numerous outbreaks were associated with raw meat consumption in several countries [9]. Indeed, a very high seroprevalence was reported in pigs, ranging from 5.2% up to 51.7% [10,11], which can affect up to 85.7% of farms [11,12] and was strictly dependent on farming features. Thereby, several reports suggested that free-range pigs are more exposed to *T. gondii*, compared to pigs raised in isolation [12,13].

The high circulation of *T. gondii* in the population of farmed animals and wildlife is widely documented, and the lack of barriers between the urban–rural environment and woodland in densely inhabited areas can favor human contact with the pathogen via livestock and wildlife [10]. Moreover, age was identified as another main factor that influences the infection rate. Indeed, older pigs are more likely to test positive for *T. gondii* than younger pigs. This factor is justified by the route of infection, as most pigs acquire *T. gondii* infection postnatally [14,15]. 

In this paper, we describe an investigation of the presence of *T. gondii* in specimens collected from home-reared pigs intended for self-consumption in order to evaluate how much home-reared swine may pose a risk to their owners. To the best of the authors’ knowledge, no information is available on *T. gondii* in pigs for domestic private farming. In Italy, especially in southern regions, the practice of raising pigs at the family level is a widespread behavior. According to national and regional legislations [16], this type of farming is strictly regulated, as owners shall hold a maximum of four pigs per household, which can be directly slaughtered at home from November to March, with respect to the European regulation on the protection of animals at the time of killing (Council Regulation (EC) No 1099/2009) under the supervision of the official veterinary services. The meat shall only be intended for self-consumption, and no reproduction is allowed. Furthermore, owners must comply with the welfare minimum standards for the protection of pigs that are confined to a farm for rearing and fattening. These conditions involve, in particular, constant watering, the hygiene of the premises, suitable flooring, and sufficient spaces for stabling and assessing any injuries, per the Legislative Decree of 7 July 2011, no. 122. Thus, official veterinary services are required to evaluate these standards when inspecting home slaughter procedures.

## 2. Methods

### 2.1. Sample Collection

During routine activities of the official veterinary services from November 2021 to March 2022 for home slaughtering, post mortem inspection and the collection of diaphragms for trichinellosis were conducted. In this context, other samples of liver, diaphragm and masseter muscle were collected, for a total of 1280 specimens from 480 pigs belonging to 305 different farms located in Napoli Province. All organs were single-sealed to avoid contamination and transferred to the Istituto Zooprofilattico Sperimentale del Mezzogiorno (IZSM) laboratories at refrigerated temperature in order to investigate the presence of *T. gondii*.

### 2.2. Real-Time PCR

Organs were homogenized by TissueLyser (Qiagen, Hilden, Germany) using 25 mg of tissue placed in 2 mL Eppendorf safe-lock tubes containing 1 mL phosphate-buffered saline (PBS) and a 4.8 mm in diameter stainless steel bead to allow mechanical lysis for 5 min at 30 Hz and subsequently centrifuged at 1650× *g* for 5 min. Next, aliquots of 200 µL of supernatant were collected from each organ homogenate, and nucleic acids extraction and purification were carried out using QIAsymphony DSP Virus/Pathogen Mini Kit (Qiagen) on QIAsymphony automated system (Qiagen), in accordance with the instructions of the manufacturer, eluted in 60 µL and stored at −20 °C until use. Elutes underwent real-time PCR using QuantStudio5 PCR thermal cycler (Thermo Fisher Scientific, Waltham, MA, USA) in a total volume of 25 µL containing 5 µL of template, 12.5 µL TaqMan 2X Universal PCR Master Mix (Thermofisher), 1 µL (12.5 µM) of each primer Toxo Forward (5′-TCCCCTCTGCTGGCGAAAACT-3′), 1 µL (12.5 µM) of primer Toxo Reverse (5′-AGCGTTCGTGGTCAACTATCGATTG -3′) and 0.5 µL (10 µM) of probe Toxo P (FAM-5′- TCTGTGCAACTTTGGTGTATTCGCAG -3′-TAMRA) [17]. The thermal profile was composed of an initial denaturation at 95 °C for 10 min, followed by 40 cycles at 95 °C for 15 s and 60 °C for 60 s for annealing and extension, respectively.

### 2.3. Droplet Digital PCR

Droplet digital PCR was performed using QX200 Droplet Digital PCR Systems (Bio-Rad Laboratories, Hercules, CA, USA) in 22 μL final volume containing 5 of template (<100 ng/μL), along with dd-PCR Supermix for Probes (Bio-Rad Laboratories) at 1× final concentration, 0.9 µM of each primer and 0.25 µM of probe and nuclease-free water to reach the final volume. The primers and probe sequences were the same used for real-time PCR. Each sample was partitioned into approximately 20,000 nL-sized droplets with AutoDG automated droplet generator (Bio-Rad Laboratories) using QXDx AutoDG Oil for Probes.

Next, the plate was sealed with pierceable foil and heat-sealed (Bio-Rad) at 180 °C using PX1 PCR plate sealer (Bio-Rad Laboratories), and PCR amplification was carried out in T100 Thermal Cycler (Bio-Rad Laboratories) with the following thermal profile: hold at 95 °C for 10 min, 40 cycles of 94 °C for 30 s and 60 °C for 1 min, 1 cycle at 98 °C for 10 min and ending at 4 °C. Finally, the plate was loaded into the QX200 Droplet Reader (Bio-Rad Laboratories) that automatically read the droplets within the wells. QuantaSoft software was used to count the fluorescent-positive and -negative droplets to calculate target DNA concentration. 

The dd-qPCR results were converted into copies/µL by multiplying the concentration obtained from the total volume of the reaction mixture (22 µL) and then divided by 5 µL, the template volume. Samples were considered to be positive when at least three droplets containing the target DNA were present, while samples were considered to be negative when no positive droplet or less than three droplets were revealed, in accordance with the instructions of the manufacturer.

### 2.4. Questionnaire 

In order to evaluate the house farm biosecurity conditions, a questionnaire was administrated to the animal keepers containing the most relevant information and what could best describe the risk factors, including the type of pigsty with the relative flooring and enclosure, the presence of wild animals nearby, the type of specific feed or if raw or cooked kitchen scraps were used for feeding, age and time of housing (Supplementary Material).

### 2.5. Data Analysis

An estimation of the true number of home-reared pigs was obtained by the evaluation of the slaughtered pigs belonging to family farms that were officially tested for trichinellosis, as, according to national regulations, 100% of slaughtered home-reared pigs must undergo *Trichinella* investigation [16]. 

In order to evaluate whether the number of our tested pigs was representative and an inferential statistic was applicable, the sample size needed to estimate the prevalence value with 95% confidence level and 4% accuracy was calculated. Thus, an expected prevalence value of 19.6% [10] and a population of 27,132, with the desired precision and confidence level for an infinite population or for a population of a specified size, was assumed to be equal to 384 pigs. These data were calculated using EpiTools online software (Epitools—Epidemiological Calculators; available online at https://epitools.ausvet.com.au/; accessed on 15 May 2023). 

Subsequently, in order to evaluate the impact of swine meat consumption belonging to these farms on the overall meat consumption in the study area, the number of slaughtered pigs related to family farms and the total slaughtered pigs belonging to intensive fattening and reproduction farms of the same area as well as imported pigs were compared. Data were obtained using the national databank VetInfo (Sistema Informativo Veterinario VetInfo; available online at https://www.vetinfo.it/; accessed on 15 May 2023) and the National Statistics Institute (Istat) database (Istituto Nazionale di Statistica (ISTAT); available online at http://dati.istat.it/Index.aspx?DataSetCode=DCSP_CONSISTENZE#; accessed on 15 May 2023).

## 3. Results

Out of 480 pigs, 3 samples tested positive for *T. gondii* by real-time PCR (0.62%). In particular, one pig showed positive results in the diaphragm, one in the masseter muscle and one in both the diaphragm and liver. An attempt at genotyping the samples that tested positive was performed using five microsatellites’ markers [18]; nevertheless, the cycle threshold (Ct) values of the real-time PCR were over 35, so no results were obtained. Furthermore, all positivity samples underwent dd-qPCR for confirmation and to evaluate the absolute quantification of *T. gondii* (Table 1).

The results of the survey administered to the farmers revealed that all the house farms had the same structural and management requirements. Specifically, they were all located in a peri-urban environment, all pigs were kept in closed masonry boxes, with biosecurity measures to guarantee no risk of contact with wild animals or rodents and cats, and no promiscuity with other animals was revealed. Animal feed was mainly composed of kitchen scraps. Moreover, although the national regulations require home-reared pigs to be slaughtered from November to March of the following year, pigs were just housed from August to December for fattening, because owners prefer to slaughter the animals before the end of the year. As a result, the pigs were only housed for 4–5 months, with an age at slaughter of less than one year (mean age of nine months). 

Furthermore, it was evaluated that the consistence of home-reared pig farms was around 17,700, but only 12,415 owned at least one pig, with an average number of 2.19 per farm (VetInfo data), for a total of 27,132.

The number of pigs slaughtered on an annual basis in the area and in the period under study was 205,646 (ISTAT data); 159,412 (77.52%) were imported, and 46,234 (22.48%) were reared and internally slaughtered, of which 41.31% (19,102/46,234) belonged to conventional fattening and reproduction intensive farms, and the remaining 58.68% (27,132/46,234) came from family farms. Thereby, home-reared pigs represented 13.19% of the total pigs that were slaughtered and intended for human consumption in the Campania region. 

## 4. Discussion

Although toxoplasmosis represents an important problem worldwide, Italy, as well as other European countries, lacks a case notification system for this disease in both humans and animals [6,19]. So far, data on the presence and prevalence of *T. gondii* in swine species are scarce or obtained from specific research conducted in restricted areas, mainly related to wild boars and/or free ranging pigs [20,21,22,23]. 

Home-reared pig farms are characterized by a particular farming method that is widely adopted in southern Italy. Owners have to comply with stringent rules, as pigs can only be used for fattening purposes, with no more than four pigs per farm, the meat can be used only for self-consumption, and the slaughter must be performed from November to March under the supervision of the Official Veterinary Authorities, who carry out a post mortem inspection and sample collection solely for trichinellosis. 

The presence of *T. gondii* is related to animal husbandry, where farming features seems to widely influence the prevalence. Indeed, extensive pig farming systems are more likely to be infected compared to intensive ones, which is mainly ascribed to environmental exposure [24,25,26]. Moreover, a study conducted in northern Italy described how the swine production category can influence the risk of acquiring the infection, where pigs farmed for reproduction compared to those for fattening showed a higher prevalence, mostly related to age at slaughter. Furthermore, biosafety levels were significantly associated with *T. gondii* infection. Thus, the more the health score increases, the more the risk of acquiring the infection decreases [19].

According to the recent literature, our results appear, surprisingly, very low compared to those of intensive farms, both housed indoors and with outdoor access [10]. Indeed, a prevalence of 0.62% was revealed. Data from other Italian regions, unfortunately, were mostly obtained through indirect methods, showing seroprevalence values that ranged from 2.1 to 3.8% in northern areas [19,20] and that were 16.14% and 10.4% in central and southern Italy, respectively [21,27]. Nevertheless, a comparison of the results obtained from different study populations and diagnostic methods could be difficult to assess [19]. In the relevant study area, no data are available on the presence of *T. gondii* DNA in intensive pig farms. However, when prevalence is assessed by direct techniques across southwestern European countries, muscle matrices reveal a 19.6% average prevalence, which may reach 47.7% in other matrices [10]. Thus, a clear difference with our results is highlighted, which is of particular relevance. 

Furthermore, it could also be hypothesized that the low infection rate may be ascribed to the age at slaughter of these pigs, as, in this unusual type of farming, the pigs are only housed for a very short period and slaughtered at a mean age of nine months or at most one year. Indeed, several studies showed that age is a relevant risk factor affecting seroprevalences in pigs, due to the typical postnatal infection that occurs in this species [14]. This difference was also reported in wild boars, where it was shown that being *T. gondii*-positive increases with age, with a 2.66-fold higher risk in 2–3-year-old pigs compared to that of 1–2-year-old pigs [15].

Thus, our results are likely to suggest that the farming conditions of the private pig farming described herein, such as the low number of animals, housing in separate boxes and limited rearing period, could mitigate parasite circulation in pigs. In addition, the present results can also be useful for inferring the low risk of transmission to humans through the self-consumption of the meat and products of animal origin that are farmed by this unconventional but extremely widespread practice in the area under study, which satisfies the very high demand for pork, with an average number of over 27,000 pigs (last 3 years’ mean) slaughtered each year. The low risk is also corroborated by the low amount of *T. gondii* DNA found by both real-time PCR and by dd-qPCR, which reflects the low absolute protozoan quantification in the edible part of the pigs, using a wide sample size to estimate the prevalence value with the desired precision and confidence levels.

However, due to the lack of data in the literature, it still remains very difficult to establish a real risk assessment of these products that are not intended for large-scale consumption.

Our data suggest that the foodstuffs of swine origin, belonging to very small farms and with a level of biosecurity dictated by the peculiarities of "family" breeding, can scarcely be exposed to contamination by infectious agents. This breeding condition, beyond satisfying an important share of the demand for pork meat, could prove to be strategic in the future to improve food safety, especially for categories of people at risk, such as pregnant women.

Further studies are, surely, needed on intensive farms in the area under study to complete the evaluation of human risk through the consumption of local pork meat as well as to integrate the data of human exposure that, to date, is extremely lacking.

## Figures and Tables

**Table 1:** Positive results obtained by real-time PCR and dd-qPCR.

Pig	Positive Matrix	Real Time PCR	dd-qPCR		
		(Ct)	Copies/µL	Copies/ Reaction	n. Positive Events
1	Diaphragm	38	0.6	2.6	10
2	Liver	37	1.1	4.8	17
	Diaphragm	38	1.4	6.2	21
3	Masseter	37	0.7	3.1	10

## Data Availability

The data presented in this study are included within the article.

## Supplementary Materials

The following supporting information can be downloaded at: https://www.mdpi.com/article/10.3390/pathogens12070882/s1.
