# Supplementary material for: Toxoplasma gondii Investigation of Home-Reared Pigs through Real-Time PCR and Digital Droplet PCR: A Very Low Prevalence
Source: Pathogens. 2023 Jun 27;12(7):882. doi: 10.3390/pathogens12070882 (PMC10384325; doi:10.3390/pathogens12070882)
Supplement: Supplementary file 1 [file pathogens-12-00882-s001.zip › pathogens-2443309-supplementary.pdf]

Survey

Farm Code: …………………..

Address: …………………..

City: …………………..

Veterinary District/Province of competence: …………………..

Context:

- ☐ Woodland environment
- ☐ Countryside
- ☐ Peri-urban
- ☐ Urban

Total N of pigs: …………………..

Total N of fences: …………………..

N of pigs in sampled pen: …………………..

Age at placement: …………………..

Duration of placement:

- ☐ 3-6 months
- ☐ > 6 months

Features:

Type of pigsty:

- ☐ masonry
- ☐ open
- ☐ open with roof
- ☐ other ………..

Fully fenced pigsties

- ☐ yes
- ☐ no

Type of flooring:

- ☐ concrete
- ☐ ground
- ☐ other …………

Presence of wild animals nearby

- ☐ yes
- ☐ no
- ☐ if yes, specify which ………...

Interaction between pets and pigs:

- ☐ yes
- ☐ no
- ☐ if yes, specify which ………...

Feeding with specific feed

- ☐ yes
- ☐ no
- ☐ if yes, specify which ………..

Feeding with kitchen leftovers:

- ☐ yes
- ☐ no
  - ○ if yes
  - ○ raw
  - ○ cooked

Date:…………….

Signature:…………….

Privacy Policy Statement
This survey is provided in respect of the privacy of its users and treats the information provided for specific research or education purposes in a confidential matter. Our Board is committed to maintaining the accuracy, confidentiality, and security of any personal information that is under its control and no sensitive data will be disclosed.
